# Fatal Influenza B Myocarditis in a 34-Year-Old Female

**DOI:** 10.5811/cpcem.2018.3.37718

**Published:** 2018-05-29

**Authors:** Taylor Dickey, Melanie Schweir, Matthew Hysell

**Affiliations:** * Michigan State University College of Osteopathic Medicine, East Lansing, Michigan; † Lakeland Health, Department of Emergency Medicine, Saint Joseph, Michigan

## Abstract

A 34-year-old female reported to the emergency department with a chief complaint of epigastric pain. Initial rapid screening was negative for both influenza A and B. The patient eventually developed myocarditis that led to pulseless ventricular tachycardia and death within 24 hours of admission. Viral smear was positive for influenza B postmortem despite the initial negative rapid screen. This case demonstrates the need for a new diagnostic criteria and treatment strategy for viral myocarditis due to influenza while concisely illustrating how the disease can progress in adults despite commonly presenting as a disease in adolescents.

## INTRODUCTION

Myocarditis can be an exceptionally challenging diagnosis to make due to its multiple etiologies, highly variable nonspecific presentations, and the lack of universal treatment standards.^1^ Therefore, it is also highly under-reported. The most common mechanism of myocarditis involves an invading virus through either the respiratory or gastrointestinal tract. These viruses infect cardiac myocytes causing an inflammatory reaction. Native macrophages and dendritic cells then form a response that triggers the release of cross-reactive self-antigens, leading to a T-cell mediated autoimmune response that further injures cardiac myocytes.^1^ Delayed cardiac manifestations such as arrhythmias, congestive heart failure, sinus tachycardia, and embolic events can occur days or even weeks after the initial viral infection.^1^ Untreated, this may eventually lead to unfavorable outcomes such as dilated cardiomyopathy, ventricular tachycardia, and sudden cardiac death.^2^

The classic culprits of viral myocarditis historically include Coxsackie virus, cytomegalovirus, and echovirus. However, there has been increasing evidence of influenza virus causing myocarditis in recent years. Based on the results of Karjalainen et al., the true incidence of myocarditis could be as high as 9%.^3^ Influenza B carries the lowest risk of developing myocarditis at 0.7% compared to type A (1.3%) and type C (3.6%).^4^ Additionally, 69% of patients who died due to an influenza B infection had some evidence of myocardial injury on autopsy report.^5^ However, most of these patients were younger than 18 years of age. Myocarditis caused by influenza B has rarely been reported in adults.^6^

## CASE REPORT

A 34-year-old female was brought to the emergency department (ED) by family with a chief complaint of severe epigastric pain. Her symptoms, which had begun five days earlier, consisted of general malaise, self-reported low-grade fevers, and a non-productive cough in addition to her epigastric pain. She had taken off work for the prior three days due to her symptoms. She reported one instance of nausea and vomiting the day prior to her ED admission. She denied any history of dysuria, hematuria, headache, or neck stiffness. Past medical history was significant for polycystic ovarian syndrome and attention deficit hyperactive disorder. Past surgical history was notable for a remote appendectomy and cholecystectomy. Social history revealed that she had quit smoking 10 years prior and drank one alcoholic beverage on average per day. She denied any recreational or intravenous (IV) drug abuse.

Triage temperature was 97.5°F, heart rate was 71 beats per minute (BPM), blood pressure measured at 136/93, respiratory rate was 20, and her oxygen saturation was 98% on room air. Approximately 20 minutes after triage, the patient remained afebrile but her heart rate had increased to 125 and blood pressure decreased to 96/56. She appeared fatigued and slightly diaphoretic. Her oropharynx was clear and moist, neck was supple with full range of motion, cardiac examination revealed no evidence of a murmur, and she displayed normal respiratory effort without any signs of distress or wheezing. Her abdomen was soft and non-tender without rebound or guarding. Urinalysis showed a specific gravity of 1.024, trace ketones, 0–2 white blood cell count per high power field (HPF), 0–2 red blood cells per HPF, and 16–20 hyaline casts. Urine pregnancy test, mycoplasmal immunoglobulin M, and influenza A/influenza B rapid screen were all negative. Chest radiograph was negative for pathology and showed a heart size and vascularity within normal limits, with clear and fully expanded lungs. Blood test results are displayed in the table below.

Fluid resuscitation was started upon arrival to the ED. Despite infusing four liters of normal saline over the course of four hours, the patient’s blood pressure never increased above a systolic pressure of 100 and she remained borderline hypotensive. Her admitting diagnosis was systemic inflammatory response syndrome (SIRS) due to a presumed viral but undetermined etiology with hypovolemia. At the time of admittance, her vital signs were a temperature of 97.5°F, a heart rate of 116 BPM, a respiratory rate of 18 breaths per minute, and a blood pressure of 94/67. She received ondansetron for nausea.

The next morning, the patient complained of worsening symptoms of malaise and weakness while denying any shortness of breath, cough, chest pain, headache, diarrhea, or anxiety. Except for her blood pressure, which had dropped to 80/50, her vitals were stable. She was given a seventh liter of fluid with modest improvement of her blood pressure, but she remained clammy and required a central line placement in the intensive care unit (ICU). Physical examination was significant for mild epigastric tenderness and acrocyanosis. At this time, she was diagnosed with dehydration secondary to severe sepsis with septic shock. She was given ceftriaxone, vancomycin, and doxycycline to cover meningococcus, methicillin-resistant *Staphylococcus aureus*, and rickettsia. Additionally, blood cultures and an electrocardiogram (ECG) were ordered and revealed questionable 0.5mm ST-segment elevation of lateral chest leads (Image). Troponin I was elevated at 0.47 ng/ml.

**CPC-EM Capsule**

What do we already know about this clinical entity?

Myocarditis is one of the more rare, but potentially fatal, complications of influenza typically seen in the adolescent population.

What makes this presentation of disease reportable?

Viral myocarditis typically presents in the adolescent population. However, in this case the patient was an adult with multiple negative diagnostic tests.

What is the major learning point?

It is essential to keep viral myocarditis on the differential in adults and even when diagnostic tests come back negative.

How might this improve emergency medicine practice?

This case points out the challenges and inefficiencies of diagnosing viral myocarditis in the emergency setting.

The patient was sent for abdominal computed tomography (CT) to look for a cause of sepsis. The imaging showed some atelectasis/bibasilar infiltrates with small bilateral pleural effusions as well as patchy enhancement of the kidneys concerning for pyelonephritis, but no significant pulmonary edema or cause of sepsis. After completing the CT, the patient decompensated into pulseless ventricular tachycardia and eventual death despite attempts at resuscitation. A postmortem influenza smear was negative for influenza A, parainfluenza A1–A4, and positive for influenza B. This finding, coupled with inflammation of the myocardium on the autopsy, led to the diagnosis of fatal myocarditis caused by influenza B.

## DISCUSSION

A review of literature revealed few confirmed, documented cases of myocarditis secondary to influenza, and even fewer cases of myocarditis specifically caused by influenza B. The case shared some common themes with the other presentations. For example, hypotension refractory to IV fluids was a common finding.^7^,^8^ Additionally, ST-segment elevations on ECG are a common theme related to myocarditis.^4^,^7^,^8^ Finally, a positive culture is a required part of diagnosing myocarditis secondary to infection.^4^,^7^,^8^ However, there were some aspects of this case that deviated from the norm . Most cases of viral infection causing myocarditis are seen in the young adult population, while our patient was 34 years old.^4^,^7^ Furthermore, influenza B is a rare cause of myocarditis compared to the more common offenders such as Coxsackie virus.

Furthering the difficulty of this diagnosis lies in accuracy of testing. Diagnosis of influenza B via reverse transcriptase polymerase chain reaction relies on a sensitivity of 54% in adults and 62% overall, which is lower than the sensitivity for influenza A (65% overall).^9^ Diagnosis of viral myocarditis is based on the Dallas criteria, which require an endomyocardial biopsy consisting of an inflammatory infiltration with necrosis and/or degeneration of adjacent myocytes.^10^ This pathologic definition has been criticized for inaccuracy in the medical community, especially considering that endomyocardial biopsies only meet these specific definitive criteria with successful diagnosis rates of 25%**.**^11^

In the treatment of influenza, oseltamivir phospate is thought to be most efficacious within 48 hours from the onset of symptoms. In patients admitted to the ICU with hemagglutinin type 1 and neuraminidase type 1 influenza, there was a reported 75% survival rate in patients given oseltamivir phospate within the time constraint (compared to 58% survival rate if left untreated).^12^ However, oseltamivir phospate administered within five days still showed some benefit in disease progression.^12^ The combination of these factors makes the diagnosis of myocarditis caused by influenza B an exceptionally difficult, yet dangerous, diagnosis to miss.

## CONCLUSION

We present an adult female with myocarditis secondary to influenza B infection. The case was complicated by low sensitivity of rapid influenza screening, inconsistent diagnosing criteria, and questionable treatment strategies. To better serve the population of patients that develop myocarditis from influenza, we need better-defined strategies to approach, diagnose, and treat myocarditis due to influenza B. Additionally, it must be recognized that, although rare, viral myocarditis should be considered in the differential diagnosis of both adults and adolescents.

Documented patient informed consent and/or Institutional Review Board approval has been obtained and filed for publication of this case report.

## Figures and Tables

**Image:**
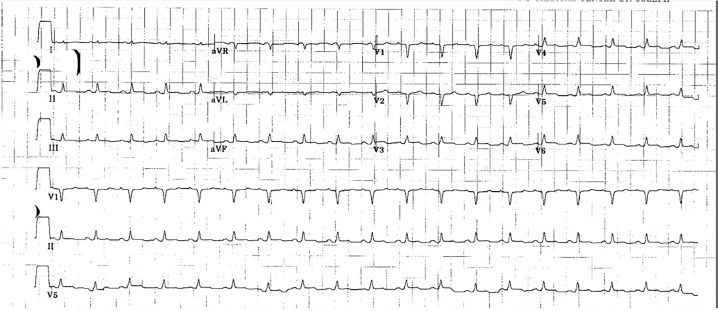
Electrocardiogram revealed mild ST-segment elevation of lateral chest leads.

**Table:** Notable components of complete blood count, blood metabolic panel, liver enzymes, and lipase.

Blood plasma, serum	Value
White blood cells	16,500/m^3^
Polymorphonuclear leukocytes	81%
Hemoglobin	19 g/dL
Platelets	287,000/mm^3^
Sodium	132 mEq/L
Potassium	4.2 mEq/L
Chloride	100 mEq/L
Bicarbonate	16 mEq/L
Blood urea nitrogen	20 mg/dL
Creatinine	1.1 mg/dL
Glucose	158 mg/dL
Aspartate aminotransferase	36 U/L
Alanine aminotransferase	24 U/L
Bilirubin	0.9 mg/dL
Lipase	33 U/L
